# Immune checkpoint inhibitor re-challenge in patients with advanced non-small cell lung cancer

**DOI:** 10.18632/oncotarget.25949

**Published:** 2018-08-17

**Authors:** Maiko Niki, Aya Nakaya, Takayasu Kurata, Hiroshige Yoshioka, Toshihiko Kaneda, Kayoko Kibata, Makoto Ogata, Shosaku Nomura

**Affiliations:** ^1^ First Department of Internal Medicine, Kansai Medical University, Shin-machi, Hirakata, Osaka 573-1010, Japan

**Keywords:** immune checkpoint inhibitor, nivolumab, pembrolizumab, re-challenge, non-small cell lung cancer

## Abstract

**Background:**

Immune checkpoint inhibitors have dramatically changed lung cancer treatment, demonstrating an overall survival benefit. There are limited data about re-challenge in patients with non-small cell lung cancer. We attempted to address this question for re-challenge of immune checkpoint inhibitor in patients with advanced non-small cell lung cancer.

**Methods:**

We retrospectively analyzed 11 patients with advanced non-small cell lung cancer treated with nivolumab and re-challenged with nivolumab/pemblorizumab at Kansai Medical University Hospital from December 2015 to December 2017.

**Results:**

Three patients achieved PR and two patients were in SD. These patients were apt to be good responders to the initial treatment, to develop immune-related adverse events and to be immediately started on re-challenge with immune checkpoint inhibitor. The median PFS was 2.7 (range, 0.5–16.1) months. Five patients (45%) had mild to moderate immune-related adverse events.

**Conclusion:**

Our study shows the effectiveness of re-challenge of immune checkpoint inhibitors in a subset of non-small cell lung cancer patients. Re-challenge might become one of treatment option for advanced non-small cell lung cancer.

## INTRODUCTION

The development of immune checkpoint inhibitors (ICPi) has dramatically changed lung cancer treatment, demonstrating an overall survival benefit. The PD-1 (programmed death-1) blocking antibodies nivolumab and pembrolizumab are currently approved for advanced non-small cell lung cancer (NSCLC). The PDL-1 (programmed death ligand-1) antibody atezolizumab was also recently approved for NSCLC. Nivolumab is the first ICPi to be approved for relapse and refractory NSCLC and has been the mainstay of treatment for NSCLC since 2015. Pembrolizumab has been approved for first-line treatment of NSCLC since 2016 in Japan. A PD-L1 expression level of >50% is essential for the use of pemblolizumab [1].

A few reports of re-challenge of ICPi have been published in patients with melanoma and involve ipilimumab monotherapy or combination therapy of ipilimumab and nivolumab [2, 3, 4, 5]. These reports revealed that re-challenge of ICPi achieved favorable results for some patients. Interestingly, some patients showed better response in re-challenge than the initial treatment. These findings suggest that re-challenge of ICPi may be a promising strategy in patients with advanced NSCLC. However, data on re-challenge in patients with NSCLC are limited.

The aims of this study were to evaluate the safety and efficacy of re-challenge of ICPi in patients with advanced NSCLC and to potentially elucidate factors that could identify patients that would gain the most benefit from re-challenge of ICPi.

## RESULTS

### Patients

The clinical characteristics of the patients (n = 11; median age, 66 years, range, 52-73 years; 82% male) included in this study are shown in Table 1. The patients were diagnosed with either squamous cell carcinoma (n=3) or non-squamous cell carcinoma (n=8). Among all patients with adenocarcinoma, patient had neither an epidermal growth factor receptor mutation and the echinoderm microtubule-associated protein-like 4-anaplastic lymphoma kinase (EML4-ALK) fusion gene. Only three patients were analyzed PDL-1 expression. Median number of previous regimens was four. The median follow-up period was 18.8(9.1-28.5) months.

**Table 1:** Patients' characteristics

Patients characteristics	n=11
Median Age, years(range)	66(52-73)
Male(n)	9
Smorking History(n)	8
Histopathorogy(n)	
Adenocarcinoma	8
Squamous	3
Driver Mutation(n)	0
ECOG performance status(n)	
0	6
1	5
Median prior chemotherapy before nivolumab(n)	4(2-7)

ECOG: Eastern Cooperative Oncology Group.

### The first treatment cycle of ICPi

All patients received nivolumab for the initial treatment of ICPi. In the initial treatment of ICPi, 5 patients (45%) achieved a partial response (PR) and 2 patients (18%) was in stable disease (SD) (Table 2). All patients were received Nivolumab for the initial treatment of ICPi. The median PFS was 4.9(0.7-18.2) months. Five in eleven patients (45%) revealed mild to moderate immune-related adverse events(irAEs). Four of five patients with irAEs achieved PR and one was in progression disease (PD).

**Table 2:** Course of immune checkpoint inhibitor therapy

			Initial treatment of ICPi						Re-challenge of ICPi			
patient number	Histopathology	PDL-1 expression(%)	Type of ICPi	irAE	Best Response	PFS (months)	Treatment between initial treatment and re-challenge	Time to re-challenge (months)	Type of ICPi	irAE	Best Response	PFS (months)
1	Ad	-	Nivo	+	SD	2.5	VNR	1.2	Nivo	+	PR	11.4
2	Sq	5	Nivo	+	PD	2.1	DTX/RT	1.6	Nivo	+	PR	16.1
3	Ad	20	Nivo	+	PR	18.2	DTX+Bev	3.9	Nivo	-	PR	5.4
4	Ad	-	Nivo	-	PR	8.9	DTX+Ram	7.9	Nivo	+	SD	3.4
5	Ad	-	Nivo	+	SD	4.9	DTX	1.0	Nivo	-	SD	2.1
6	Ad	-	Nivo	-	PD	0.7	DTX	3.9	Nivo	+	PD	3.5
7	Sq	-	Nivo	-	PR	9.4	DTX+Ram	4.7	Nivo	-	PD	1.6
8	Ad	-	Nivo	-	PD	0.8	DTX/nab-PTX/S-1/GEM	12.7	Nivo	-	PD	0.5
9	Sq	-	Nivo	+	PD	1.4	nab-PTX	4.2	Nivo	-	PD	1.1
10	Ad	-	Nivo	-	PR	11.7	DTX/RT	4.6	Nivo	-	PD	1.2
11	Ad	10	Nivo	+	PR	13.6	-	6.1	Pem	+	PD	2.0

ICPi: immune checkpoint inhibitor, PFS: progression free survival, irAE: immune-related adverse event, Ad: adenocarcinoma, Sq: squamous cell carcinoma, PR: partial response, SD: stable disease, PD: progression disease, VNR: vinorelbine, DTX: docetaxel, RT: radiation therapy, Bev: Bevacizumab, Ram: Ramucirumab, nab-PTX: nab-paclitaxel, S-1: Tegafur/Gimeracil/Oteracil, Gem: gemcitabine, Nivo: nivolimab, Pem: pembrolizumab.

### Treatment between the first ICPi and second ICPi

Ten patients (91%) were received cytotoxic chemotherapies between the initial ICPi and re-challenge treatment. One patient was not received any treatment. Eight in ten patients were given docetaxel based regimens and two patients were received radiation. Median duration from initial ICPi to re-challenge of ICPi was 4.2 (1.0-12.7) months.

### The re-challenge of ICPi

In the re-challenge of ICPi, ten patients were administered nivolumab and one was administered pembrolizumab. Among the total 11 patients, 3 patients (27%) achieved a PR and 2 patients (18%) were in SD (Table 2). The median PFS was 2.7 (0.5–16.1) months. Five patients (45%) had mild to moderate irAEs. Two of the five patients with irAEs achieved PR, one was in SD and two were in PD. Three of the five patients who had developed irAEs in the initial treatment developed immune-related adverse events(irAEs) in the re-challenge.

### Frequency of AEs and irAEs

The frequencies of adverse events(AEs) and irAEs are shown in Table 3. The most common AEs (any grade) in the initial treatment were appetite loss (n=3) and diarrhea (n=2), fatigue (n=2), and rash (n=2). The most common AEs (any grade) in the re-challenge were appetite loss (n = 4) and fatigue (n= 3), and rash (n=2). Severe AEs (grade > 3) were not seen in both courses.

**Table 3:** Frequency of adverse events and immune-related adverse events

a. Adverse Events				
n=11	Initial Treatment		Re-challenge	
	Any Grade	Grade3-4	Any Grade	Grade3-4
Fatigue	2	0	3	0
Diarrhea	3	0	1	0
Appetite loss	3	0	4	0
Nausea	1	0	2	0
Vomiting	0	0	1	0
Constipation	1	0	1	0
Stomatitis	1	0	0	0
Skin Rash	2	0	2	0
Edema	1	0	1	0
Liver dysfunction	1	0	1	0
Pneumonia	1	0	0	0
Glucose intolerance	0	0	1	0

b. Immune-related adverse events		
	Immune-related adverse events	any grade(n)
skin	rash	4
hepatic	liver dysfunction	2
endocrine	glucose intolerance	1
gastro-intestinal	diarrhea	4
	mucositis	1
pulmonary	interstitial lung disease	1
others	edema	1

The most common irAEs were rash (n=4) and diarrhea (n=4) and liver dysfunction (n=2).

Severe irAEs (grade > 3) were not seen in both courses. There was no patient who discontinued treatment due to AE or irAEs.

## DISCUSSION

ICPi have dramatically changed lung cancer treatment over the past years, demonstrating an overall survival benefit. The question of re-challenge with the same therapeutic approach regularly occurs in daily practice. We attempted to address this question for ICPi in patients with advanced NSCLC.

In our study, five in eleven patients (45%) achieved PR or SD, which suggests that a re-challenge of ICPi might be a promising treatment. However, the question occurs as to in which patients would gain best benefit from re-challenge.

One of the parameter which reflects the efficacy of re-challenge could be the response to the initial treatment with ICPi. In the report of re-challenge of ICPi in patients with melanoma, six in eight patients who responded well in the initial treatment revealed good response to the re-challenge [6]. In our cohort, four of five patients who had responded to the initial treatment responded to the re-challenge. Only one patient (case2) was PD in the initial treatment and he achieved PR in re-challenge. He was received chemotherapy and radiation before the re-challenge. Thus, ‘abscopal effect’ might have occurred by radiation. ‘Abscopal effect’ is the theory about cancer antigen presentation by radiation. The radiation therapy to some tumors activates whole body anti-tumor immunity, and non-irradiated cancer cells also become smaller. There are three non-randomized trials concerning to ‘abscopal effect’ in patient with melanoma [7, 8, 9]. In these trials, patients were received both radiation and ipilibumab. 10-27% of patients developed ‘abscopal effect’, and 13-23% of patients were in SD. Recently, a secondary analysis of patients with metastatic NSCLC from the phase I pembrolizumab trial, KEYNOTE-001, showed that patients who had previously received radiotherapy had significantly longer PFS and overall survival than those who did not [10]. Although the precise mechanism is unknown, radiation might induce antitumor immunity and bring a better response. However, there is another patient who was received radiation between the treatments (case10). This patient did not achieved PR in re-challenge. Thus, ‘abscopal effect’ might not have occurred in this patient. The difference between case 2 and case 10 was different duration to re-challenge. Case 2 was 1.6 months, whereas, case 10 was 4.6 months. Furthermore, case 2 developed irAEs in the initial treatment, whereas, case 10 did not. Randomized studies in ipilibimab in melanoma have shown that patients with irAEs have high response rates and excellent clinical outcome with observation alone [11, 12]. We previously reported that irAEs might be a prospective factor of nivolumab efficacy in patients with NSCLC [13]. Four in six patients (67%) who developed irAEs in the initial treatment achieved PR or SD and three in five patients (60%) in the re-challenge achieved PR or SD. It suggests that patients who developed irAEs might achieved better outcome than patients without irAEs. Thus, the presence of irAEs during the initial treatment might be a predictive factor of efficacy of re-challenge of ICPi.

In the duration between the two ICPi treatments, eight of the ten patients received docetaxel-based regimens. An important factor in the use of cytotoxic anticancer drugs is immunogenic cell death (ICD), in which the cytotoxic anticancer drug or radiotherapy elicits an immune response leading to cell death of the tumor cells [14, 15, 16, 17]. Some anti-cancer agents are reported to induce ICD [18–28]; for example, cyclophosphamide [18, 19, 20], doxorubicin [21, 22, 23], mitoxantrone [24, 25, 26] and oxaliplatin [15, 27, 28] are considered to induce ICD. However, we could not find any report of ICD induced by docetaxel and our results failed to reveal that the interval treatment regimens impacted the effect of re-challenge.

Although the length of time required to build the immune response to ICPi remains unclear, we speculate that the establishment of acquired immunity could require more time than building of spontaneous immunity. Thus, the duration from the initial treatment to re-challenge might be important. In the responders to the re-challenge, the median duration from the initial treatment to the re-challenge was 1.6 months, whereas, non-responder was 4.7 months. This result reveals that the duration between the initial treatment to the re-challenge might be concerned to the effectiveness of re-challenge. We assumed that the building of immune response might have started by the initial treatment and still kept building even after the initial treatment failure. Therefore, when the re-challenge starts, the building of the immune response might have been completed and immune response might occur promptly. However, after some interval without ICPi, the immune response might become reset, thus the response might be worse after long interval drug withdrawal. Our data suggests that it may be feasible to restart the re-challenge within three months from the last of the initial treatment.

The expression of PD-L1 influence the therapeutic effect of ICPi [29]. Positivity of PD-L1 is the most effective status for efficacy of ICPi. However, in our cohort, not all patients could be analyzed PDL-1 expression because of the condition of the patients, tumor sites, or insurance limitations. Therefore, in our cohort, only three patients were analyzed it. It was too small size to make a conclusion about the relationship between PDL-1 expression and effect of ICPi.

This study has some limitations, including its retrospective design, a single facility, and small sample. Statistical analysis could not be done due to the small number of cases. Since the basic experiment has not been carried out, the detailed mechanism is unknown. Furthermore, we did not use immune-related response criteria because this was not a prospective trial and most physicians were not yet familiar with such criteria. We were also unable to examine the expression of PD-L1 before treatment for all patients because of the limitation described above.

In conclusion, our findings suggest that re-challenge of ICPi is tolerable and safe. Further, re-challenge may be a promising treatment in some patients who responded well in the initial treatment or who developed irAEs in the initial treatment, and the shorter duration to re-challenge may bring better response to re-challenge. Thus, these patients should be considered for re-challenge. However, additional studies are needed to identify patient populations most likely to gain the best benefit from re-challenge of ICPi and to clarify the precise mechanisms of anticancer immune response.

## PATIENTS AND METHODS

We conducted a retrospective study of patients with advanced NSCLC who were treated with single-agent nivolumab after platinum failure and were received re-challenge of nivolumab/pembrolozumab from December 2015 to December 2017 at Kansai Medical University Hospital, Osaka, Japan. The initial nivolumab (3 mg/m^2^) was administered every 2 weeks until the occurrence of disease progression, or unacceptable toxicity. After cessation of the initial treatment of nivolumab, patients were received nivolumab (3 mg/m^2^, evert 2 weeks) or pembrolizumab (200 mg/body, every 3 weeks) as re-challenge until the occurrence of disease progression, unacceptable toxicity, withdrawal, or death. This study was conducted in accordance with the Declaration of Helsinki and the requirements of the institution's review board.

### Study assessments

Treatment responses of the patients were evaluated according to the Response Evaluation Criteria in Solid Tumors using whole-body computed tomography performed every 8 to 12 weeks. However, because of the possibility of pseudo-progression in patients considered to have progressive disease, tumor size was carefully evaluated with reference to the Guidelines for the Evaluation of Immune Therapy Activity in Solid Tumors [30]. The evaluation of toxicity was based on the Common Toxicity Criteria for Adverse Events, version 4.0 [31]. AEs were evaluated for 12 weeks. irAEs were defined as rash, diarrhea, colitis, thyroid disorder, hepatitis, arthritis, and other conditions [32]. Progression-free survival (PFS) was defined as the time from the start of initial ICPi treatment to objective disease progression.
